# Cardiotoxicity of Anthracyclines

**DOI:** 10.3389/fcvm.2020.00026

**Published:** 2020-03-18

**Authors:** Daniela Cardinale, Fabiani Iacopo, Carlo Maria Cipolla

**Affiliations:** ^1^Cardioncology Unit, European Institute of Oncology, IRCCS, Milan, Italy; ^2^Cardiology Division, European Institute of Oncology, IRCCS, Milan, Italy

**Keywords:** cardiotoxicity, anthracyclines, early detection, troponin, prevention, reversibility, ACE-inhibitors, beta-blockers

## Abstract

Cardiotoxicity is a feared side effect that may limit the clinical use of anthracyclines. It may indeed affect the quality of life and survival of patients with cancer, regardless of oncological prognosis. This paper provides an overview of anthracycline-induced cardiotoxicity in terms of definition, classification, incidence, risk factors, possible mechanisms, diagnosis, and treatment. We also report effective strategies for preventing cardiotoxicity. In addition, we discuss limiting current approaches, the need for a new classification, and early cardiotoxicity detection and treatment. Probably, anthracycline-induced cardiotoxicity is a continuous phenomenon that starts from myocardial cell injury; it is followed by left ventricular ejection fraction (LVEF) and, if not diagnosed and cured early, progressively leads to symptomatic heart failure. Anthracycline-induced cardiotoxicity can be detected at a preclinical phase. The role of biomarkers, in particular troponins, in identifying subclinical cardiotoxicity and its therapy with angiotensin-converting enzyme inhibitors (mainly enalapril) to prevent LVEF reduction is a recognized and effective strategy. If cardiac dysfunction has already occurred, partial or complete LVEF recovery may still be obtained in case of early detection of cardiotoxicity and prompt heart failure treatment.

## Introduction

Anthracyclines are cytostatic antibiotics (1), introduced into the clinical field in the 1960s. As of 2012, anthracyclines were among the most diffused chemotherapeutic agents, and they still represent the base of treatment in many solid cancers and hematological malignancies (1, 2).

Unfortunately, anthracyclines are considered the principal culprit drugs behind chemotherapy-induced cardiotoxicity (1–5). The pathognomonic manifestation of anthracycline-induced cardiotoxicity is a hypokinetic cardiomyopathy progressively leading to heart failure, first described in 1967 (6). The onset of anthracycline-cardiomyopathy, also at the pre-clinical stage, may negatively affect the cardiovascular outcome of patients as also limit the chemotherapeutic strategies (4, 5).

## Incidence And Risk Factors

The risk of anthracycline-induced heart failure increases as the cumulative dose administered increases: 3–5% with 400 mg/m^2^ and as high as 18–48% at 700 mg/m^2^ (4). However, there is a different level of risk for each patient scheduled for anthracycline therapy: patients less than 5 years old or more than 65 years old, with prior or concurrent chest irradiation, pre-existing heart disease, or already known cardiovascular risk factors, have an increased risk for cardiotoxicity

(Table 1) (4, 7). Moreover, anthracycline-induced cardiotoxicity risk increases with the use of other agents that may increase its incidence. In particular, trastuzumab, while very effective in treating breast cancer, interferes with myocyte survival pathways, crucial in countering the toxic effects of anthracyclines (5, 7, 8).

**Table 1:** Baseline risk factors for anthracycline-induced cardiotoxicity (4, 7).

**Current myocardial disease**	**Demographic and other** **CV risk factors**
• Heart failure • Asymptomatic LV dysfunction (LVEF <50%) • Evidence of CAD (previous myocardial infarction, angina, PCI or CABG, myocardial ischemia) • Moderate and severe VHD with LVH or LV impairment • Hypertensive heart disease with LV hypertrophy • Hypertrophic cardiomyopathy • Dilated cardiomyopathy • Restrictive cardiomyopathy • Cardiac sarcoidosis with myocardial involvement • Significant cardia arrhythmias (AF, ventricular tachyarrhythmias)	• Age (<5 or >65 years) • Family history of premature CV disease (<50 years) • Arterial hypertension • Diabetes mellitus • Hypercholesterolemia
**Previous cardiotoxic cancer treatment**	**Lifestyle risk factors**
• Prior anthracycline use • Prior radiotherapy to chest or mediastinum	• Smoking • High alcohol intake • Obesity • Sedentary habit

*AF, atrial fibrillation; CABG, coronary artery bypass graft; CAD, coronary artery disease; CV, cardiovascular; LV, left ventricular; LVEF, left ventricular ejection fraction; LVH, left ventricular hypertrophy; PCI, percutaneous coronary intervention; VHD, valvular heart disease*.

## Mechanisms

The specific mechanisms of anthracycline cardiotoxicity still remain unclear. A potential mechanism is the generation of reactive oxygen species (ROS), changes in iron metabolism, and Ca2þ signaling. In 2014, topoisomerase (Top) 2β was indicated as the critical mediator of anthracycline's cardiac toxic effect (9). Top2 can uncoil deoxyribonucleic acid (DNA) filaments during DNA replication, transcription, or recombination. The anthracycline inhibition of Top2β causes mitochondrial dysfunction and leads to activation of cell death pathways and ROS deposit (2, 3, 10).

**Figure 1:**
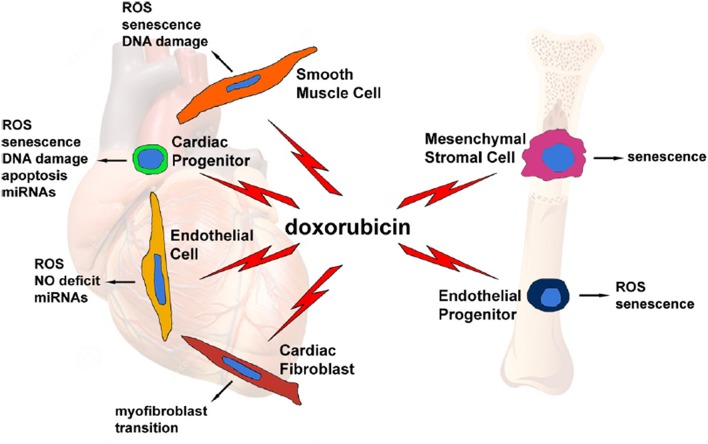
Graphical representation of several doxorubicin-targeted cell types, with potential side effects and cellular and molecular events evoked by the drug. From Cappetta et al. (11).

The cardiomyocyte has always been considered the main cellular target of anthracycline toxic effect in the heart, as their destruction results in the progressive development of cardiac dysfunction. More recently, however, other cell types—such as cardiac progenitor cells, cardiac fibroblasts, and endothelial cells—have been identified as potential additional targets, creating a more complex and intriguing scenario in the pathogenesis of anthracycline-induced cardiomyopathy (Figure 1) (11). So far, the principal mechanisms, with potential differential impact and grade of involvement in different cell types, are oxidative stress, DNA damage, senescence, and cell death.

## Classification

A previous and more dated classification identified three distinct types of anthracycline-induced cardiotoxicity (Table 2): acute, occurring after a single dose, or a single course, with the onset of symptoms within 14 days from the end of treatment, which is usually reversible; early-onset chronic, occurring within 1 year, the principal form of cardiotoxicity, from a clinical and epidemiological stand-point, presenting as a dilated-hypokinetic cardiomyopathy, with progressive evolution toward heart failure; and late-onset chronic, developing years, possibly decades, after the end of anthracycline therapy. The two chronic forms are considered irreversible, with a poor prognosis and a limited to heart failure therapy. This classification stems back to early 1980s, and it is mainly based on small retrospective studies reporting the occurrence of heart failure symptoms in childhood cancer survivors (12–14). In particular, in a milestone study, Steinherz et al. reported cases of heart failure occurrence many years after the end of anthracycline-chemotherapy, and the percentage of patients with cardiac dysfunction, as well as the severity of the dysfunction itself, increased in parallel with time elapsed from the end of anthracycline administration (14). However, the clinical relevance of such a classification at present is uncertain, especially when referred to adult populations.

**Table 2:** Old classification of anthracycline-induced cardiotoxicity (7, 12–14).

**Characteristics**	**Acute** **cardiotoxicity**	**Early-onset chronic** **cardiotoxicity**	**Late-onset chronic cardiotoxicity**
Onset	During or within 2 weeks after AC treatment	Within 1 year after the completion of AC treatment	>1 year after the completion of AC treatment
Dose dependent	Unknown	Yes	Yes
Clinical features	Depression of myocardial contractility	Dilated/Hypokinetic cardiomyopathy	Dilated/Hypokinetic cardiomyopathy
Course	Usually reversible	Usually irreversible	Usually irreversible
		Refractory to traditional heart failure therapy	Refractory to traditional heart failure therapy
		Poor prognosis	Poor prognosis

**Figure 2:**
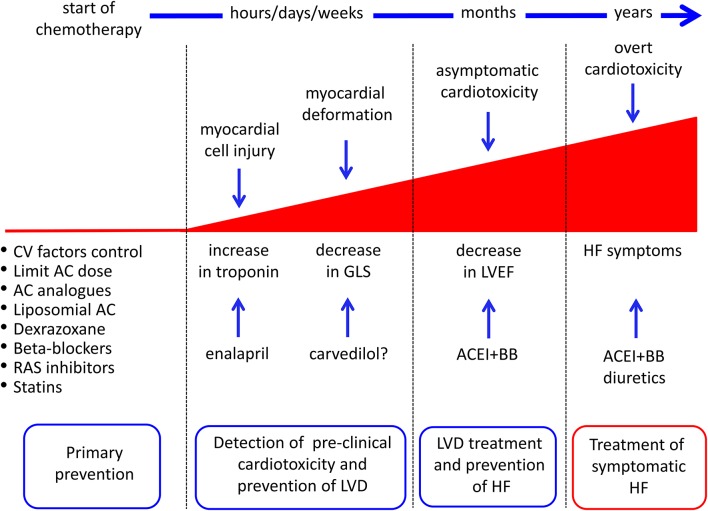
Possible strategies for cancer drug-induced cardiotoxicity detection, prevention, and treatment. AC, anthracyclines; ACEI, angiotensin-converting enzyme inhibitors; BB, beta-blockers; CV, cardiovascular; GLS, global longitudinal strain; HF, heart failure; LVD, left ventricular dysfunction; RAS, renin-angiotensin system. From Cardinale et al. (8).

In particular, recent findings challenge this old classification, suggesting that anthracycline-induced cardiotoxicity is potentially a continuous phenomenon, starting at the myocardial cell level, followed by progressive functional decline, progressively leading to overt heart failure. (Figure 2) (5, 8, 15). To be practical, anthracycline-associated cardiotoxicity is now thought to occur at the time of first exposure, a hypothesis supported by the finding of troponin release after anthracycline administration (16). Clinical presentation may occur years later the initial damage (16–18). Looking at symptoms, the diagnosis may take years (“late” cardiotoxicity). Considering LVEF reduction, it may take months (“early” cardiotoxicity). With the use of circulating biomarkers, such as troponin (pre-clinical myocardial cell damage), prompt identification of cardiotoxicity is possible, allowing for an “acute” form. So far, we are probably observing the evolving stages of the same phenomenon and not three distinct diseases (15, 17, 18).

## Diagnosis and Definition

The diagnosis of anthracycline-induced cardiotoxicity has remained the same over the last 60 years. It has always been based on heart failure symptoms, and, later, also on evidence of LVEF drop (echocardiography or multi-gated acquisition scans) (4, 18). A former definition adopted was an LVEF absolute decrease higher than 10% points, associated with a decline <50% (5). More recently, the consensus [Plana et al. (19)] defined it as an LVEF decrying >10% points, with a final value <53% (19). In patients at low risk—i.e., without risk factors or a negative cardiovascular history, with an indication to receive a low dose of anthracyclines (total cumulative dose ≤ 240 mg/m^2^) or standard dose followed by trastuzumab-based regimens—cardiac monitoring is not suggested by the American Society of Clinical Oncology guidelines. Moreover, they suggest a diagnosis of cardiotoxicity based on clinical symptoms (20). Reasons comprise “medicalization, the possibility of causing stress and anxiety, and costs” to be incurred (20, 21). Otherwise, the international cardiological guidelines recommend monitoring of cardiac function by serial LVEF measurements, but do not provide an accurate indication on timing, frequency, modalities, and long-term schedule (7). Moreover, a diagnosis based on symptoms or asymptomatic decrease of LVEF is not only delayed, but also potentially prevents any form of effective prevention, as the cardiac damage may be no longer reversible (17, 18).

**Figure 3:**
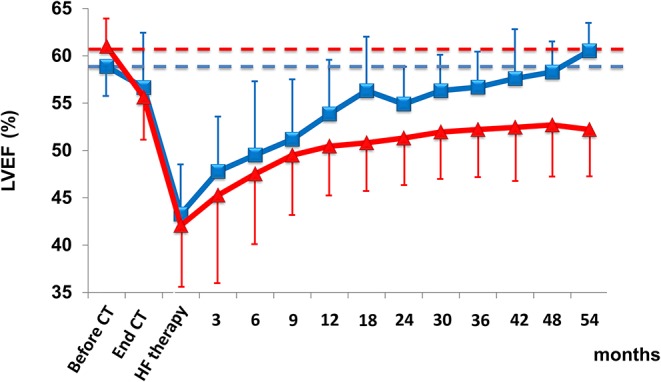
LVEF in patients with cardiotoxicity and with partial (triangle) or full (square) recovery with heart failure therapy. Data are mean ± SD. CT, chemotherapy; HF, heart failure. From Cardinale et al. (15).

A recent study evaluating a significant (*n* = 2,625) population scheduled for anthracycline therapy showed that close monitoring of LVEF after chemotherapy allowed nearly all (98%) cases of cardiotoxicity to be identified within the first 12 months of follow-up (15). In addition, early treatment with angiotensin-converting enzyme (ACE)-inhibitors (enalapril) and beta-blockers (carvedilol or bisoprolol) enabled normalization of cardiac function in most cases (82%), but only 11% of patients who had renormalized LVEF had full recovery—i.e., the same LVEF value as before the start of anthracyclines—while the final LVEF value in 71% of patients remained below the baseline value (Figure 3).

These findings confirm that this approach is limited in identifying reversible cardiotoxicity, probably because left ventricular compensation mechanisms have been exhausted (8). Of great importance, the evidence of a normal LVEF does not exclude the risk of future deterioration of cardiac function.

## Treatment

The historical concept that anthracycline-induced cardiotoxicity is irreversible, with a reported mortality rate up to 60% within 2 years of diagnosis, is now reconsidered. In particular, this belief is based on seminal studies in which heart failure therapeutic strategies were limited (i.e., digoxin, diuretics), or on studies with small populations, retrospective design, short follow-up, or on case reports (22–30).

Up until 2010, the response to heart failure therapy of patients with anthracycline-induced cardiotoxicity hadn't been thoroughly investigated. Moreover, these kind of patients have been excluded from large randomized trials evaluating the impact of current heart failure therapies (8).

**Figure 4:**
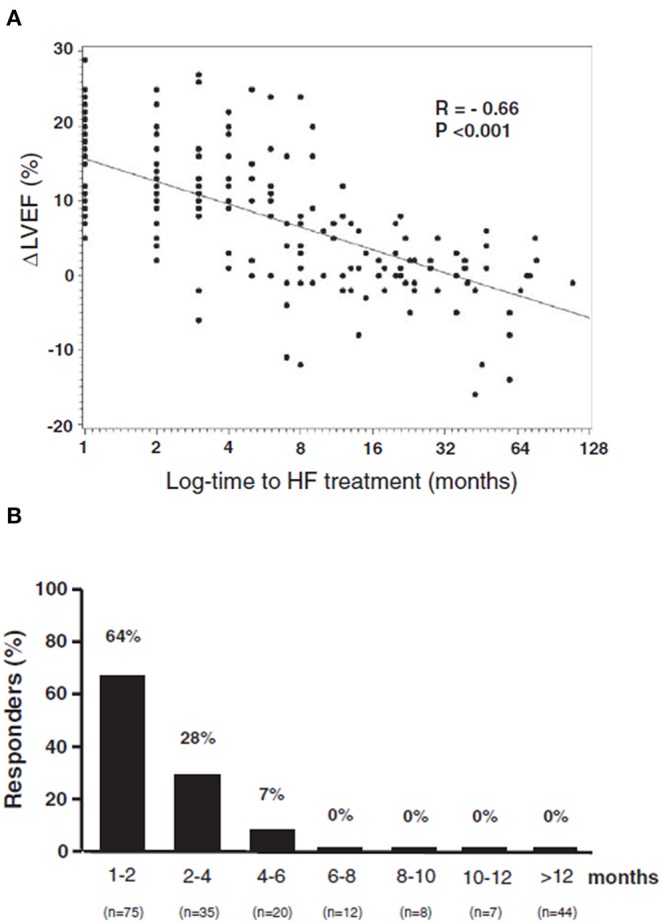
**(A)** Percentage of patients who recovered (Responders), according to the time elapsed from anthracycline administration and the start of heart failure therapy. **(B)** Relationship between maximal LVEF during the follow-up period and log time elapsed from chemotherapy and the start of treatment [time-to-heart failure (HF) treatment]. From Cardinale et al. (31).

The effectiveness of ACE-inhibitors and beta-blockers has been prospectively assessed in two extensive papers (15, 31). In 201 patients with anthracycline-induced cardiotoxicity, an inverse relationship in terms of LVEF improvement has been found between the time interval from the end of chemotherapy and the beginning of heart failure therapy (Figure 4A) (31). LVEF recovery rate was 64% in those treated early (i.e., within 2 months after the end of chemotherapy); later on, however, this percentage rapidly decreased, with no complete recovery after 6 months. After 12 months, obtaining even partial LVEF improvement was almost impossible (Figure 4B) (31). It emerges that cardiotoxicity is not irreversible, but that reversibility is a matter of time, depending on early diagnosis, allowing prompt treatment. Furthermore, these findings, based on standard cardiac symptoms surveillance, might miss this change (8).

On the contrary, close monitoring and timely treatment with HF therapies have reported that they are critical for functional recovery in a non-selected population treated with anthracycline, allowing early detection of cardiotoxicity in the vast majority of cases during the first year after chemotherapy, with normalization of LVEF (final value of LVEF >50%) in 82% of cases (15). However, only 11% of patients had a complete restoration (i.e., final LVEF equal to baseline). This highlights the need for detection methods able to identify early cardiotoxicity and for strategies aimed at preventing the development and the progression of left ventricular dysfunction.

## Preclinical Early Detection

Today, at an early preclinical stage, we can detect cardiotoxicity long before symptoms of heart failure occur and before an asymptomatic drop in LVEF. Most data relate to cardiac biochemical markers: mainly troponins and echocardiography of tissue Doppler and strain (5, 7, 8).

### Troponin Assessment in Anthracycline-Treated Patients

Troponin may be considered the gold standard biomarker for myocardial injury and cardiotoxicity from different causes/etiologies (32). Troponin has many advantages: elevated cardiac specificity, high sensitivity, availability, and costs respective to imaging methods. Moreover, there are limited variability issues. In this field, several studies have demonstrated that troponins may detect cardiotoxicity in patients treated with anthracyclines (Table 3) (33–56).

**Table 3:** Clinical studies demonstrating Troponins as predictor of anticancer drug-induced left ventricular dysfunction (33–56).

**Study (year)**	**Patients (n.)**	**Cancer type**	**Drugs**	**Troponin type**	**Cut off**	**Timing of assessment**
Lipshultz et al. (33)	15^*^	ALL	AC	T	0.03 ng/mL	Before CT; 1–3 days after each dose
Cardinale et al. (34)	201	Various	HD CT	I	0.04 ng/ml	0–12–24–36–72 h after CT
Cardinale et al. (35)	232	Breast cancer	HD CT	I	0.04 ng/ml	0–12–24–36–72 h after CT
Auner et al. (36)	30	Hematological	HD Cycl	T	0.03 ng/ml	Before CT; 1–14 days after CT
Sandri et al. (37)	179	Various	HD CT	I	0.04 ng/ml	0–12–24–36–72 h after CT
Cardinale et al. (38)	703	Various	HD CT	I	0.04 ng/ml	0–12–24–36–72 h after CT
Specchia et al. (39)	79	Hematological	AC	I	0.15 ng/ml	Before CT; weekly x 4 times
Kilickap et al. (40)	41	Various	AC	T	0.10 ng/ml	Before CT; 3–5 days after 1st and last dose
Lee et al. (41)	86	Hematological	AC	I	0.20 ng/ml	Before each dose
Schmidinger et al. (42)	74	Renal cancer	Sunitinib/sorafenib	T	0.02	Before CT, bimonthly, symptoms occurrence
Cardinale et al. (43)	251	Breast cancer	AC, TRZ	I	0.04 ng/ml	Before and after each cycle
Sawaya et al. (44)	43	Breast cancer	AC+taxanes+TRZ	HS-I	0.015 ng/ml	Before CT; after 3 and 6 months during CT
Lipshultz et al. (45)	205^*^	ALL	AC/AC+dexrazoxane	I/T	Any detectable amount	Before CT; 1–7 days after each dose; end CT
Sawaya et al. (46)	81	Breast cancer	AC+taxanes+TRZ	HS-I	30 pg/mL	Before CT; after 3 and 6 months during CT
Draft et al. (47)	53	Various	AC	I	0.06 ng/ml	Before CT; after 1, 3, 6 months
Mornos et al. (48)	74	Various	AC	HS-T	NA	Before CT; after 6, 12, 24, 52 weeks
Mavinkurve-Groothuis et al. (49)	60^*^	ALL	AC	HS-T	0.01 ng/mL	Before CT; after 3 and 12 months
Ky et al. (50)	78	Breast cancer	AC+taxanes+TRZ	HS-I	NA	Before CT; after 3 and 6 months during CT
Mornos et al. (51)	92	Various	AC	HS-T	NA	Before CT; after 12 and 36 weeks
Putt et al. (52)	78	Breast cancer	AC+taxanes+TRZ	HS-I	NA	Before CT; every 3 months (max 15 months)
Zardavas et al. (54)	412	Breast cancer	AC+taxanes+TRZ	HS-T/US-I	14 ng/L/40 ng/L	Before CT; week 13, 25, 52; month 18, 24, 30, 36
Olivieri et al. (54)	99	Lymphoma	AC/lipoAC	US-I	0.08 ng/ml	Before CT; 1, 24–72 h after each cycle
Kitayama et al. (55)	40	Breast cancer	AC/AC+TRZ/TRZ	HS-T	NA	Before CT; every 3 months during CT
Shafi et al. (56)	82	Breast cancer	AC	US-I	NA	1, 24 h after each cycle

AC, anthracycline-containing chemotherapy; ALL, acute lymphoblastic leukemia; CT, chemotherapy; Cycl, cyclophosphamide; HD, high-dose; LAP, lapatinib; lipoAC, liposomial anthracycline; NA, not available; I, troponin I; T, troponin T; TRZ, trastuzumab; HS, high-sensitive; US, ultra-sensitive

*, *pediatric population*.

The most extensive study included 703 cancer patients, in whom Troponin I (TnI) was assessed before and during the first 72 h after chemotherapy (early TnI), and after 1 month (late TnI) (38). Three different troponin release patterns were recognized: Troponin I remained within the normal interval in 72% of patients, rose at only early evaluation in 21%, and increased at early and late assessments in 9%. Patients with no rise in troponin showed little difference in LVEF and had a good prognosis, with a low incidence of significant adverse heart events (MACE) (1%) during follow-up. Alternatively, TnI-positive patients had a higher rate of MACE: In particular, severe cardiac dysfunction and a higher rate of MACE were associated with a persistent TnI elevation compared to patients with only a temporary rise (*p* < 0.001). Based on the high negative predictive value (99%), TnI has been able to safely identify low-risk patients, limiting the need and subsequent costs of close long-term cardiac monitoring (34, 35, 38). Conversely, TnI-positive patients deserve more stringent monitoring, mainly those showing a persistent TnI increase.

In summary, we can assert that troponin evaluation in patients treated with anthracyclines allows for:

1. Prediction of the development of future left ventricular dysfunction;
2. Prediction of left ventricular dysfunction severity, because the peak value of troponin is closely related to the extent of LVEF reduction;
3. Stratification of cardiac risk after anthracyclines and tailoring of the schedule of post-chemotherapy monitoring of cardiac function;
4. Identification of cardiotoxicity prone patients, in whom a cardioprotective therapy can be considered; and
5. Exclusion of most patients from prolonged cardiologic surveillance.

On the other hand, the identified weakness points are:

1. Repeated assessments of troponins are needed to detect positivity;
2. The ideal timing for troponin detection must still be defined;
3. Standardization of routine troponin use in this clinical setting is a current need; and
4. Timing in which a single sampling of troponin could be obtained.

### Other Circulating Biomarkers

Although patients with pre-treatment levels of natriuretic peptides (BNP and N-terminal prohormone) tend to experience cardiac events (including cardiac dysfunction), the results are sparse (44, 57, 58).

More recently (57), BNP levels were shown to be significantly higher after every anthracycline cycle in subjects following cardiac events, while another study demonstrated an association between an increased BNP at 72 h after chemotherapy and a decrease of LVEF at 1 year (59).

Actually, studies of other biomarkers, including microRNAs (miRNAs), C-reactive protein (CRP), growth differentiation factor-15 (GDF-15), myeloperoxidase (MPO), and galectin-3 (Gal-3) have not demonstrated an association between pretreatment biomarker levels and cardiovascular outcomes (60, 61).

Regarding monitoring for cardiovascular toxicity during therapy, CRP has shown conflicting findings (50).

More recently, some reports emerged in the field of microRNAs, in particular for miR-1, showing a trend to earlier detection of cardiotoxicity respective to troponin (62, 63).

Another study of patients during a period of 10 years after anthracycline therapy did not find an association with Gal-3 and LV dysfunction.

A separate study that included Gal-3 and ST2 found no association with these biomarkers and LVEF 1 year after therapy (64, 65).

### Tissue Doppler and Strain Echocardiography

Novel echocardiographic methods have emerged as sensitive parameters in the early identification of cardiotoxicity. In particular, introduction of tissue Doppler and strain imaging techniques can detect early subclinical changes in cardiac function, before LVEF falls (4, 7, 19, 51). In this respect, myocardial deformation (strain imaging) has emerged as a sensitive marker for earlier detection of myocardial dysfunction. In particular, 2D (and more recently, 3D) speckle tracking imaging, allowing the evaluation of global myocardial deformation in the longitudinal axis (global longitudinal strain, GLS, %), has become a clinical standard. Several papers demonstrated the value of GLS in detecting subclinical myocardial dysfunction, with prognostic relevance in terms of overt LV dysfunction in cancer patients (66–69).

The recent ASE/EACVI consensus defined a relative decrease in GLS of >15% from baseline as an indicator of subclinical LV dysfunction and appropriate use criteria for multi-modality imaging include strain for the evaluation of patient candidates for chemotherapy (19, 70). Finally, the SUCCOUR trial (first randomized controlled trial of GLS-guided therapy introduction) will better define the role of GLS for surveillance for chemotherapy-related cardiac dysfunction (71).

However, these methodologies are not always readily available in all laboratories and seldom used in the routine evaluation of patients receiving anthracyclines (8).

### An Integrated Approach to Biomarkers and Cardiac Imaging

Breakthroughs in laboratory technology have allowed for the introduction of more specific and sensitive troponin assay methods (55), which are able to measure minimal amounts (high-sensitivity [HS] dosing systems) of a biomarker that were not detectable with previous methods. This is of pivotal importance, since troponin release as a consequence of anthracycline cardiotoxicity may be minimal, and it is essential to use high-precision dosing systems (72).

The first HS troponin trial enrolled 45 breast cancer patients who were treated with anthracyclines, taxanes, and trastuzumab (44). International and regional myocardial function was assessed at baseline, every 3 months, with tissue Doppler and strain imaging, combined with troponin. A reduction in the longitudinal strain and an increase in HS troponin were predictive of late left ventricular dysfunction after the end of anthracyclines. Notably, the combined assessment of imaging methods and changes in troponin resulted in an increased specificity (93% combined vs. 73% for each single method). Ky et al. tested a multi-marker approach in a similar population of breast cancer patients receiving the same anti-cancer therapy regimen (50). All levels of the markers increased significantly from baseline (except for NT-proBNP and Galectin-3). However, at the end of anthracycline therapy, only HS troponin absolute values and changes in troponin and myeloperoxidase levels resulted as predictors of further development of left ventricular dysfunctions.

## Primary Prevention: Reduction of the Direct Cardiotoxic Effect (Figure 2)

### Limitation of the Maximum Dose of Anthracyclines

Present oncologic guidelines recommend limiting the total cumulative dose of anthracyclines to 450–550 mg/ml (4, 8). However, this may limit the effectiveness of anti-cancer treatment. Moreover, significant variability exists in terms of proneness to anthracycline cardiotoxicity, suggesting that genetic variation might modulate the risk (5, 7, 8).

### Use of Less Cardiotoxic Anthracycline Analogs

Epirubicin, idarubicin, and mitoxantrone are analogs of anthracyclines that are less cardiotoxic than conventional anthracyclines. Epirubicin cardiotoxicity occurs after higher doses of doxorubicin. However, to obtain the same clinical response, higher doses must be given. In preclinical studies and animal models, idarubicin and mitoxantrone also showed a less cardiotoxic profile than doxorubicin (5, 7).

### Use of Liposomal Anthracyclines

In the heart, liposomes cannot get out from the vascular space because capillaries have tight junctions. As the tendency to accumulate in the heart cells is limited, this may reduce the risk of cardiotoxicity. On the contrary, the liposomes reach high concentrations in the tumor site, leaving the circulatory system where tumor growth damages the capillaries itself (73, 74).

## Primary Prevention: Pharmacologic Prevention (Figure 2)

### Lifestyle Measures

Before pharmacologic strategies, primary prevention starts indeed with lifestyle corrective measures.

Since a strong link exists between cancer and cardiovascular risk factor, addressing smoking and sedentary habits (potentially leading to obesity, with a detrimental role especially in the post-menopausal women), as well as high alcohol intake, is pivotal. A healthy diet has been associated with a protective effect in terms of cancer relapses and cardiovascular disease, while smoking has an ominously detrimental effect. While light to moderate alcohol intake has shown a protective impact in terms of cardiovascular disease, the results in terms of risk of developing cardiotoxicity are conflicting (75–77).

Of notice, several pieces of evidence emerged on the protective role of exercise training (and eventually, cardiac rehabilitation) against cardiotoxicity (78).

### The Use of Cardioprotection

The use of cardioprotective drugs to reduce the direct cardiotoxic effect is a potential alternative to anthracycline treatment modifications, dosage limitations, or interruptions (4, 5, 8).

The hypothesis that iron chelators may reduce the cardiotoxicity induced by anthracyclines suggests that dexrazoxane may be a clinically useful cardioprotective agent (9, 79). Doxorubicin is a potent Top2 inhibitor. In the clinical scenario, many studies demonstrated that dexrazoxane significantly reduces cardiotoxicity in adults and pediatric populations: Patients treated with dexrazoxane had a significantly lower incidence of heart failure than untreated patients. Apart from patients with metastatic breast cancer treated with doses of doxorubicin >300 mg/mq and despite previous findings, dexrazoxane is not routinely used in clinical practice, because suspected of interfering with the anti-tumor effects and by the occurrence of secondary malignancies. In September 2011, the outcome of a referral (80) that recommended several restrictions on dexrazoxane use in both children and adults with cancer was published. However, several new trials on the benefit-risk of dexrazoxane have been published from then (81–83). So far, dexrazoxane results an effective cardioprotector when administered with anthracycline chemotherapy being not associated with a reduction in anti-tumor efficacy or survival or a relevant increased risk of second primary malignancies, and can be recommended as a cardioprotector particularly for children and adolescents for whom the development of anthracycline-induced cardiotoxicity could have a crucial prognostic impact. These studies contributed to the CHMP's decision to remove the contraindication on Cardioxane (84).

Macedo et al. recently published a systematic review and meta-analysis of nine trials (seven randomized and two retrospective non-randomized trials) on the efficacy of dexrazoxane in patients with breast cancer treated with anthracyclines (with or without trastuzumab). Despite the quality of available evidence remaining low, dexrazoxane was shown to reduce the risk of heart failure and cardiac events, independently from previous exposure to anthracyclines. The oncological response and survival rates were not affected by dexrazoxane (85).

Other potentially cardioprotective agents have been studied in animal models and small clinical studies. Preliminary data are promising, but they need to be ratified by further extensive studies (2, 5, 7, 8).

### The Use of Cardiovascular Agents

Several heart failure drugs have been shown to be effective in terms of cardioprotection against anthracylines (Table 4) (86–98).

**Table 4:** Cardiovascular drugs showing a prophylactic effect against anticancer therapy-induced LVD in adult cancer populations.

**Study (year)**	**Study design/follow-up**	***N***	**Cancer type**	**Drugs**	**Intervention**	**Results**
**BETA-BLOCKERS**						
Kalay et al. (86)	RCT/6 months	50	Various	AC	Carvedilol	No LVEF↓
Kaya et al. (87)	RCT/6 months	45	Breast cancer	AC	Nebivolol	No LVEF and NT-proBNP↑
Seicean et al. (88)	Retrospective/5 years	318	Breast cancer	AC,TRZ	Beta-blockers	HF ↓
Pituskin et al. (89)	RCT/12 months	99	Breast cancer	CT+TRZ	Bisoprolol	No LVEF ↓
**ACEI**						
Cardinale et al. (90)	RCT/12 months	114	Various	HD CT	Enalapril	No LVEF ↓; MACE incidence ↓
Pituskin et al. (89)	RCT/12 months	99	Breast cancer	CT+TRZ	Perindopril	No LVEF ↓
**ARB**						
Nakamae et al. (91)	RCT/7 days	40	NHL	AC	Valsartan	No LVEDD↑; no BNP and ANP↑; no QT↑
Cadeddu et al. (92)	RCT/18 months	49	Various	AC	Telmisartan	No peak strain rate ↓; no interleukin-6↑
Gulati et al. (93)	RCT/1.5–16 months	120	Breast cancer	AC+Tx+TRZ	Candesartan	No LVEF ↓
**ALDOSTERONE ANTAGONISTS**						
Akpek et al. (94)	RCT/6 months	83	Breast cancer	AC	Spironolactone	No LVEF↓; no TNI and BNP↑;
**ACEI** **+** **BETA-BLOCKERS**						
Bosh et al. (95)	RCT/6 months	90	Hematological	AC	Enalapril + carvedilol	No LVEF↓; death↓; HF ↓
**STATINS**						
Acar et al. (96)	RCT/6 months	40	Hematological	AC	Atorvastatin	No LVEF↓
Seicean et al. (97)	Retrospective/5 years	67	Breast cancer	AC	Statins	No HF ↓
Chotenimitkhun et al. (98)	PO	51	Various	AC	Atorvastatin/simvastatin	No LVEF↓

*ACEI, angiotensin-converting enzyme inhibitor; ANP, atrial natriuretic peptide; ARB, angiotensin receptor blocker; BNP, brain natriuretic peptide; HD CT, high-dose chemotherapy; LVD, left ventricular dysfunction; LVEF, left ventricular ejection fraction; LVEDD, left ventricular end-diastolic diameter; HF, heart failure; MACE, major adverse cardiac events; NHL, non Hodgkin lymphoma; NT-proBNP, N-terminal-proBNP; QT, QT interval; PO, prospective observational; RCT, randomized controlled trial; Tx, taxanes; TNI, troponin I; TRZ, trastuzumab*.

Overall, a recent meta-analysis of randomized clinical trials of adult patients that underwent chemotherapy and cardiovascular therapies vs. placebo with follow-up (17 trials, 1,984 patients) showed higher (although with small changes) LVEF values at follow-up in cancer patients receiving neurohormonal therapies (99).

#### Beta-Blockers

The non-cardioselective beta-blocker carvedilol is cardioprotective against anthracyclines toxicity. *In vitro* studies and a small randomized clinical trial, the drug was able to prevent the development of ventricular dysfunction (86). In breast cancer patients, carvedilol blunted strain abnormalities and the increase in troponin, preserving diastolic function, after anthracycline use (100). However, the drug failed to prevent an LVEF reduction >10% (101). It appears that carvedilol's efficacy is linked to its antioxidant activity rather than its beta-blocking action. Indeed, a comparative study of carvedilol and atenolol, a selective β1 antagonist with no antioxidant properties, showed that carvedilol—but not atenolol—prevented mitochondrial damage and mitigated the ultrastructural changes associated with doxorubicin (8, 102).

Nebivolol, a selective β1 antagonist with vasodilatory properties, started 7 days before anthracyclines and continued for 6 months in 27 patients with breast cancer prevented a significant decrease of LVEF and an increase of NT-proBNP (87). In a retrospective study including 106 breast cancer patients, a reduced incidence of heart failure over a 5-year follow-up period was associated with the continuation of beta-blocker therapy during oncology treatment—including anthracyclines (88). Existing data indicate, from preclinical studies, that cardio-specific beta blockers offer superior protection against anthracycline damage than non-cardioselective ones (8).

#### ACE-Inhibitors and Sartans

Experimental data demonstrated a crucial role of the renin-angiotensin system (RAS) in the development and progression of cardiomyopathy induced by anthracyclines (90). Valsartan, administered in combination with anthracyclines, blunted natriuretic peptides increase, the increase in chamber size in patients with non-Hodgkin's lymphoma treated doxorubicin (91). The authors hypothesized a direct inhibition of the drug, independent from hemodynamic effects (8, 90).

Telmisartan, started before epirubicin, was able to prevent strain reduction and inflammatory markers increase because of its RAS blocking action, but also because of its anti-inflammatory and anti-oxidant properties (92).

In the PRADA (Prevention of Cardiac Dysfunction during Adjuvant Breast Cancer Therapy) trial candesartan—but not metoprolol—administrated with adjuvant chemotherapy including anthracyclines, with or without trastuzumab, can protect against an early decline in LVEF, assessed with cardiac MRI (93).

The MANTICORE-101 study (Multi-disciplinary Approach to Novel Therapies in Cardiology Oncology Research) tested the use of perindopril vs. bisoprolol in the prevention of left ventricular remodeling, defined as an increase in end-diastolic diameters and primary study point, and of left ventricular dysfunction in HER2+ breast cancer patients treated with trastuzumab prior to anthracycline (89). Neither drug prevent left ventricular remodeling; however, the use of both drugs was associated with a preserved left-ventricular function in multi-variate analysis.

The combination of enalapril and carvedilol have been tested in the OVERCOME study (preventiOn of left-ventricular dysfunction with enalapril and carvedilol). The study involved 90 patients treated with anthracyclines, with malignant hemopathies. LVEF didn't change in the intervention group after 6 months, but decreased significantly in controls. In addition, the intervention group had a lower rate of combined death or heart failure or death, heart failure, and a final LVEF of <45 % (95).

#### Aldosterone Antagonists

A recent randomized trial, including 43 breast cancer patients, evaluated the use of spironolactone vs. placebo. Spironolactone was started 1 week before anthracyclines. Three weeks after the end of chemotherapy, the treated group did not show relevant variations in LVEF and rise in troponin I and NT-proBNP (94). In ELEVATE (Effect of Eplerenone on Left Ventricular Diastolic Function in Women Receiving Anthracyclines for Breast Cancer), a recent randomized placebo-controlled trial, administration of eplerenone for 6 months was not associated with significant differences in ventricular function compared with placebo in patients with breast cancer treated with anthracyclines (103).

#### Statins

The effect of statins on cardiotoxicity of anthracyclines is most likely due to their pleiotropic effect, and in particular, to their antioxidant properties (8). Forty hematologic cancer patients with no history of heart disease were randomized to receive atorvastatin or placebo before the onset of anthracyclines (95). The dosage was 40 mg/day, regardless of the levels of cholesterol, and lasted for 6 months. During the follow-up, a reduction of the high-sensitivity reactive C protein level and no significant changes in LVEF were observed in the statin group.

Conversely, the LVEF value in the control group resulted in a significant reduction from the baseline. The protective effect of statins also emerged when chemotherapy was started in patients already receiving statins for the prevention of cardiovascular disease (96). In a retrospective observational study of 67 breast cancer patients treated with anthracycline, statin therapy continued to be associated with significant reduction in the risk of heart failure and cardiac-related mortality during follow-up. More recently, patients on statin therapy for the prevention of cardiovascular disease reported a smaller drop in LVEF at 6 months in a retrospective observational study, including 51 patients with breast cancer or hematological malignancies treated with anthracyclines (97).

#### Perspectives

A recent study identified the molecular and cellular signature of dose-dependent, doxorubicin-mediated cardiotoxicity and provided evidence that prokineticin receptor (PKR-1)-1, acting at myocardial and vascular level, is a promising target to combat cardiotoxicity of cancer treatments (104).

Since G protein-coupled receptors (GPCRs) are a target of 40% of clinically used drugs and newly identified cardioprotective agents that bind GPCRs of adrenalin, adenosine, melatonin, ghrelin, galanin, gpelin, prokineticin, and cannabidiol may further aid in the cardioprotective task (105).

## Prevention in Selected High-Risk Patients

Prevention may be an option for all patients who are candidates for cardiotoxic therapy (primary prevention) or restricted to patients with preclinical symptoms of cardiotoxicity, with the advantage of limiting prophylactic therapy to a small number of patients (also reducing the side effects of preventive therapy, i.e., hemodynamic effects) (Figure 2).

**Figure 5:**
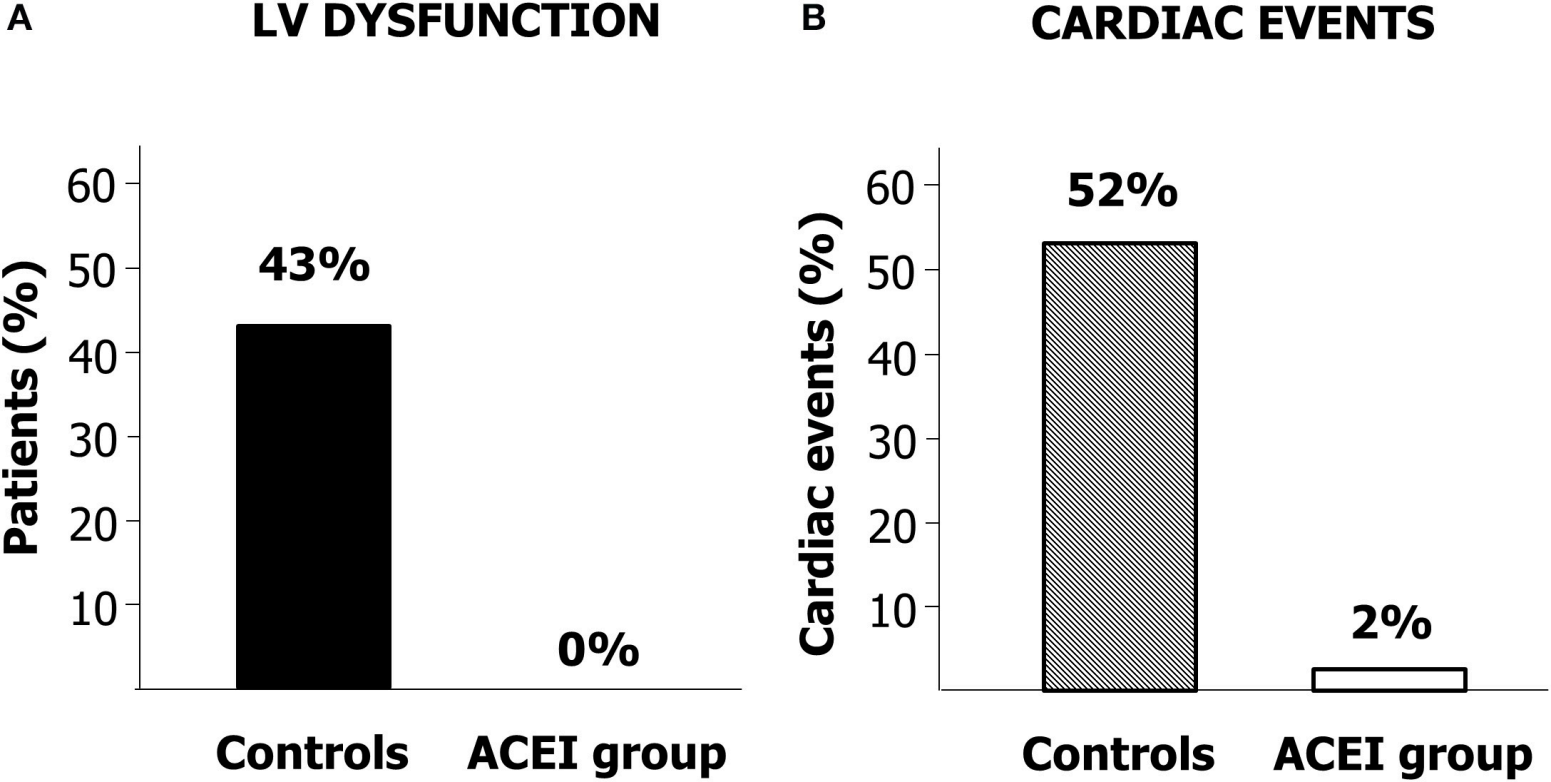
**(A)** Percentage of patients developing cardiac dysfunction in the enalapril-treated group (ACEI Group) and controls. **(B)** Incidence of cardiac events in patients treated with ACEI Group and in Controls. Modified from Cardinale et al. (90).

A randomized trial has tested the cardioprotective capacity of enalapril, involving 473 patients with different types of cancers treated with high-dose chemotherapy (90). 114 patients showed an increase in troponin and were randomized for treatment with or without enalapril. After the end of chemotherapy, enalapril was begun, titrated as tolerated, and continued for 1 year. No patients in the enalapril-treated group showed a decrease in LVEF by 10 absolute points below the value of 50%—the study's primary endpoint—and the incidence of major cardiac events was remarkably small (Figure 5). Of note, in the enalapril community, the LVEF value was still the same as the baseline value in 80% of cases after a follow-up duration of 12 months, showing that enalapril can be a very effective drug in the complete preservation of systolic function in this population.

Two studies are currently evaluating the efficacy of carvedilol as a preventive therapy in a selected patient with a deterioration in the strain parameter. For Research NCT02177175 (Carvedilol for the Prevention of Anthracyclines/Anti-HER2 Therapy-Associated Cardiotoxicity between Women with HER2+ breast cancer Using Myocardial Strain), the primary endpoint is the identification of a reduced LVEF value during the 1-year follow-up. At Northwestern University of Chicago, the research is still hiring.

## Primary vs. Secondary Prevention

Enalapril, which began early after the increase in troponin during anthracycline chemotherapy and continued for 12 months, is an effective therapy to avoid left ventricular dysfunction and subsequent heart events (90). Repeated assessment, however, is needed to detect an increase in troponin, as the marker may increase at different times after infusion with therapy (dose of anthracycline and schedules). Primary prevention, applied to all anthracycline-treated patients, does not pose this downside. The ICOSONE (International CardioOncology Society-One) randomized trial prospectively compared the efficacy of two different approaches, to test whether enalapril, initiated in all patients before chemotherapy (Prevention Group), was able to prevent troponin rise and further the development of left ventricular dysfunction, and to test whether this strategy was more successful than enalapril initiated only after troponin elevation during chemotherapy (Troponin-triggered Group) (106). The study included 273 patients from 21 different Centers of Oncology. The most-often administered anthracyclines were epirubicin and doxorubicin. During chemotherapy and the 12-month follow-up, no significant reduction in LVEF and a minimal incidence of cardiovascular events were detected in both groups. Only three patients experienced cardiotoxicity defined as a 10% reduction in LVEF, below 50% value.

In brief, the main result of the study was that the two approaches appear to be similarly effective in preventing left ventricular dysfunction and adverse cardiac events, endorsing the use of enalapril in averting anthracycline-induced cardiotoxicity, irrespectively from the strategy used.

Which strategy is best? Secondary prevention (i.e., troponin driven) has the limitation of repeated blood samplings. Nevertheless, considering the high negative predictive value of troponin (34, 35, 37, 38), this strategy appears warranted and cost-effective, as it permits the exclusion of low-risk patients (patients without troponin rise, the vast majority) from long-term monitoring programs based on imaging techniques with a relevant cost–benefit ratio by reducing “medicalization, distress, anxiety, and costs” (21). Primary prevention, although not needing a repeated evaluation of troponin during chemotherapy, can be hard in terms of clinical surveillance during the drug up-titration to include 100% of patients. Finally, it may expose to potential side effects all those low-risk subjects for cardiotoxicity (Table 5) (106).

**Table 5:** Pros and Cons of primary prevention vs. secondary prevention with enalapril (83).

**Primary prevention** **with enalapril**	**Enalapril in troponin + patients**
PROS: • Very low incidence LVD & MACE • Troponin assessment not required	PROS: • Very low incidence LVD & MACE • Monitoring during up-titration in about 20% pts • Only pts at high-risk exposed to side effects • FU monitoring not required in troponin negative patients • Low cost-benefit ratio
CONS: • Monitoring during up-titration in 100% • All pts exposed to side effects • FU monitoring required in all pts • High cost-benefit ratio	CONS: • Repeated TNI assessment

*LVD, left ventricular dysfunction; MACE, major adverse cardiac events; FU, follow-up; TNI, troponin I*.

## Conclusion

Anthracycline-induced cardiotoxicity is still a significant problem that compromises the quality of life and overall survival of cancer patients. However, recent findings demonstrate that this form of cardiomyopathy is mostly reversible with early detection and prompt therapeutic introduction strategy. Probably, anthracycline-induced cardiotoxicity is a single and continuous phenomenon, from cellular to clinical stage, starting with myocardial cell injury, followed by progressive LVEF decline and, potentially, overt heart failure. The current standard for monitoring cardiac function (periodic assessment of LVEF), detects cardiotoxicity at a late stage when a significant impairment has already occurred, precluding the chance of effectively prevent and treat its development.

The use of troponins to identify patients with subclinical cardiotoxicity combined with early treatment with ACE-inhibitors occurrence appears to be an effective method to prevent anthracycline-related left ventricular dysfunction and cardiac events.

Finally, adoption of internal procedures, shared in a multi-disciplinary team, may actively aid in optimizing patient management. In this respect, a direct relationship with the laboratory medicine service for the assessment of troponin values during chemotherapy and the availability of a cardiologist and a dedicated nurse staff should always mix with an active collaboration with the referral oncologist/hematologist (possibly, surgeon) for updates and remains of pivotal importance (107, 108).

## Author Contributions

DC, FI, and CC contributed conception, design of the review, and wrote the first draft. All authors contributed to manuscript revision, read, and approved the submitted version.

### Conflict of Interest

The authors declare that the research was conducted in the absence of any commercial or financial relationships that could be construed as a potential conflict of interest.
